# Machine Learning in the Prediction of Venous Thromboembolism: Systematic Review and Meta-Analysis

**DOI:** 10.2196/77339

**Published:** 2025-12-23

**Authors:** Ruyi Ma, Weifeng Yu, Jian Tian, Yunyan Tang, Hua Fang, Xin Ming, Hua Liu

**Affiliations:** 1 School of Nursing, Jilin University, Changchun, China; 2 China-Japan Union Hospital of Jilin University, Jilin, Changchun, 130033, China, 86 13271865951; 3 School of Mathematics, Jilin University, Changchun, China

**Keywords:** artificial intelligence, machine learning, venous thromboembolism, venous thrombosis, prediction

## Abstract

**Background:**

With the increasing use of machine learning (ML)–based risk prediction models for venous thromboembolism (VTE) in patients, the quality and applicability of these models in practice and future research remain unknown. The prediction mechanism of ML and the number of selected factors have been research hotspots in VTE prediction.

**Objective:**

This study aimed to systematically review the literature on the predictive value of ML for VTE.

**Methods:**

PubMed, Web of Science, MEDLINE, Embase, CINAHL, and Cochrane Library databases were searched for studies published up to March 26, 2025. Studies that developed and validated an ML model for VTE prediction in the patient population and were published in English were eligible, and studies with duplicate data were excluded. The Prediction Model Risk of Bias Assessment Tool was used to assess the risk of bias in the included studies. Meta-analyses were performed to evaluate the C-index, sensitivity, and specificity.

**Results:**

A total of 27 studies with 596,092 patients reported the assessment value of ML models for predicting VTE. The risk of bias assessment yielded 18 (67%) studies with a high risk of bias, 8 (30%) with an unclear risk of bias, and 1 (4%) with a low risk of bias. The pooled sensitivity and specificity were 0.79 (95% CI 0.78-0.80) and 0.82 (95% CI 0.81-0.82), respectively. The positive likelihood ratio was 5.02 (95% CI 3.81-6.60), the negative likelihood ratio was 0.27 (95% CI 0.22-0.33), and the diagnostic odds ratio was 20.14 (95% CI 13.69-29.63; *P*<.001). A random-effects model was leveraged for meta-analysis of the C-index, which was 0.84 (95% CI 0.80-0.88). The most significant predictors for VTE were age, D-dimer level, and VTE history.

**Conclusions:**

ML has been shown to effectively predict VTE in patients. However, a high risk of bias was identified in most of the included studies (18/27, 67%), primarily due to shortcomings in handling missing data and reporting the study design. Consequently, future research must prioritize external validation and address methodological rigor to facilitate the translation of these models into routine clinical practice.

## Introduction

Venous thromboembolism (VTE) is a common vascular disease characterized by the formation of blood clots in the veins. As the third most common cardiovascular condition globally, VTE affects up to 5% of the population worldwide [1]. VTE includes pulmonary embolism (PE) and deep vein thrombosis (DVT). In Europe, VTE accounts for approximately 544,000 fatalities annually [2]. At least 1 in 12 middle-aged adults will develop VTE during their remaining lifetime in the USA [3]. In Asia, the incidence of VTE ranges from 14 to 57 per 100,000 person-years [4]. VTE has become a significant global health concern.

VTE is frequently asymptomatic, underscoring the importance of early prevention and intervention prior to clinical manifestation. Epidemiological evidence indicates that the risk of VTE is multifactorial [5], necessitating a comprehensive assessment to guide clinical decision-making.

Currently, thrombosis assessment tools, such as the Caprini risk assessment model and the Padua predictive score, are used to assess VTE in patients. However, these tools exhibit limited adaptability to complex patient data and fail to dynamically assess the risk of VTE occurrence in patients. Advances in computational technologies and the emergence of mobile health have accelerated the integration of machine learning (ML) in predictive disease modeling [6]. ML can address these gaps through nonlinear modeling techniques, tackling challenges that traditional statistical methods cannot solve and improving predictive accuracy. Many studies have developed prediction models for VTE based on ML methods have been published, especially in recent years [7,8]. However, few systematic reviews have evaluated these existing ML models. Therefore, we performed a systematic review and meta-analysis to evaluate the predictive performance of ML in VTE risk prediction, aiming to provide evidence-based support for the development of high-accuracy prediction tools and offer more comprehensive insights for future clinical applications.

## Methods

### Study Registration

This study adhered to the PRISMA (Preferred Reporting Items for Systematic Reviews and Meta-Analyses) guidelines and was prospectively registered on PROSPERO (CRD420251041604). The detailed PRISMA checklist is presented in Checklist 1. Ethics approval was not required because this systematic literature review focused on retrospective studies.

### Search Strategy

PubMed, Web of Science, MEDLINE, Embase, CINAHL, and Cochrane Library databases were systematically searched until March 26, 2025. The search strategy involved controlled terms and free-text terms, with no restrictions on geographical location or publication year. Details of the search strategy are provided in Multimedia Appendix 1.

### Eligibility Criteria

We developed detailed inclusion and exclusion criteria for our systematic review based on language, study type, model construction, and outcome measures, as detailed in Textbox 1.

Textbox 1. **Inclusion and exclusion criteria for original studies.**

**Inclusion criteria**

- Language: only original studies published in English
- Study type: case-control, cohort, nested case-control, case-cohort, and cross-sectional studies
- Model construction: comprehensive construction of machine learning (ML) models for predicting venous thromboembolism
- Outcome measures: receiver operating characteristic, C-index, true positive (TP), true negative (TN), false positive (FP), false negative (FN), sensitivity, specificity, accuracy, positive predictive value, and negative predictive value. In the absence of reported TP, TN, FP, or FN, these were calculated from known variables (sensitivity, specificity, and accuracy)

**Exclusion criteria**

- Language: non-English language original studies
- Study type: studies categorized as meta-analyses, reviews, guidelines, conferences, case reports, editorials, or expert opinions
- Model construction: studies with only risk factor analysis but without construction of a complete ML model; no predictive models were constructed using ML algorithms
- Outcome measures: none of the following outcomes were reported: receiver operating characteristic, C-index, TP, TN, FP, FN, sensitivity, specificity, accuracy, positive predictive value, and negative predictive value

### Study Selection and Data Extraction

The retrieved studies were imported into EndNote. After removing the duplications, 2 researchers (JT and XM) independently screened the titles and abstracts to select the studies based on the inclusion criteria. Subsequently, the full texts were screened to determine the final inclusion. Any discrepancies were resolved by a third reviewer (HL). The level of agreement between the 2 researchers was assessed using the Cohen κ statistic.

We extracted data from the included studies using a standardized evidence table based on the CHARMS (Critical Appraisal and Data Extraction for Systematic Reviews of Prediction Modeling Studies) checklist [9]. The following data were extracted: author, year of publication, country, study design, study population, sample size, predicted outcome, training cohort, testing cohort, measure calibration, and external validation. Data extraction was carried out independently by 2 researchers (RM and YT). Any discrepancies were resolved by consulting a third researcher (HL) to reach an agreement.

### Risk of Bias Assessment

PROBAST (Prediction Model Risk of Bias Assessment Tool) was used to assess the risk of bias in the eligible studies. PROBAST includes 4 domains: participants, predictors, outcomes, and analysis, which reflect the overall risk of bias and usability. Each domain contains 2 to 9 questions. There are 3 answers (yes or probably yes, no or probably no, and no information) to each question. A domain is regarded as high risk if any question is answered with no or probably no. To be considered low risk, all questions in the domain should be answered as yes or probably yes. The overall risk of bias is rated as low when all domains are deemed to be low risk, and the overall risk of bias is rated as high when at least 1 domain is deemed to be high risk. Two authors (RM and WY) independently assessed the risk of bias using the PROBAST. Disagreements were discussed and resolved by a third author (HF).

### Statistical Analysis

We used Review Manager (version 5.3) and MetaDisc software for statistical analysis. *I*^2^ was used to describe the percentage of variability in effect estimates due to heterogeneity. The following metrics were used: 0%-25% (low heterogeneity), 25%-50% (moderate heterogeneity), 50%-75% (substantial heterogeneity), and 75%-100% (considerable heterogeneity). Given the differences in the included variables and inconsistent parameters among the ML models, random-effects models were prioritized in the meta-analysis of the C-index.

We performed a meta-analysis of sensitivity and specificity using a diagnostic tool that reconstructed fourfold tables, including true positive (TP), false positive (FP), true negative (TN), and false negative (FN) findings. For studies lacking direct reporting, fourfold tables were calculated using reported metrics, such as sensitivity, specificity, accuracy, and the total patient population. Furthermore, studies in which other key statistics, such as the C-index, were missing and could not be obtained from the authors or reliably calculated were excluded from the meta-analysis to safeguard the validity of our findings. Deeks funnel plot was used to assess potential publication bias.

## Results

### Study Identification

**Figure 1.**
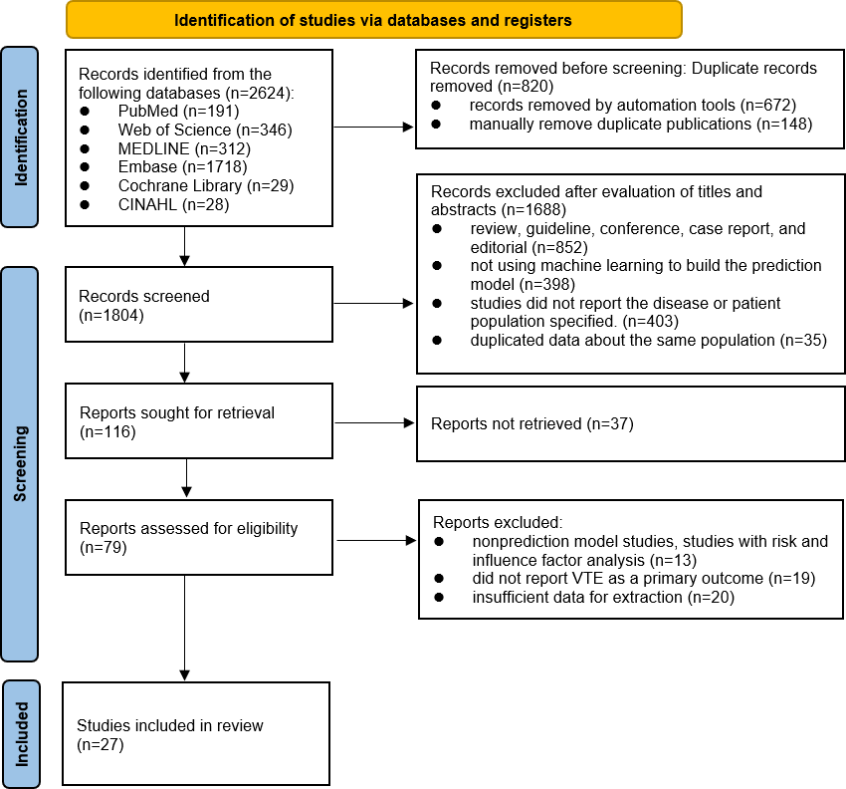
The PRISMA (Preferred Reporting Items for Systematic Reviews and Meta-Analyses) flow diagram for study selection. VTE: venous thromboembolism.

Figure 1 shows the literature selection and filtering process following the PRISMA 2020 guidelines. A total of 2624 papers were identified from 6 databases: PubMed (n=191, 7.3%), Web of Science (n=346, 13.2%), MEDLINE (n=312, 11.9%), Embase (n=1718, 65.5%), Cochrane Library (n=29, 1.1%), and CINAHL (n=28, 1.1%). After excluding 820 (31.3%) duplicates (identified by the software: n=672, 81.9%; manually: n=148, 18%), 1688 (64.3%) articles were excluded by screening the titles and abstracts, and 1804 (68.8%) records were screened. The primary reasons for exclusion were unrelated publication types (n=852, 50.5%), studies that did not use ML for prediction model development (n=398, 23.6%), studies that focused on nonrelevant diseases or conditions (n=403, 23.9%), and studies that reported duplicate patient populations (n=35, 2.1%). Of the 116 records sought for retrieval, 37 (31.9%) were not obtained. Finally, 79 (68.1%) reports were assessed for eligibility through full-text reviews. We excluded studies that focused on risk or influencing factor analysis without developing a predictive model (n=13, 16%), studies that did not assess VTE as an outcome (n=19, 24%), and studies with data that could not be extracted for analysis (n=20, 25%). Ultimately, 27 (34%) studies met the inclusion criteria and included 596,092 patients. The level of agreement between the researchers (JT and XM) was reflected by a Cohen κ of 0.78, which represents good agreement.

### Characteristics of the Included Studies

The characteristics of the included studies are summarized in Table 1. The years of publication of the articles ranged from 2019 to 2024, and 21 (78%) of 27 were published in 2022 or later. Of the 27 included studies, the predicted conditions were PE (n=3, 11%), DVT (n=9, 33%), VTE (n=14, 52%; studies did not clearly distinguish between PE and DVT), and peripherally inserted central catheter–related thrombosis (n=1, 4%).

**Table 1.** Summary of study characteristics of the 27 included publications on machine learning−based venous thromboembolism prediction.

Author	Country	Study design	Population	Outcome to be predicted	VTE^a^ cases/sample size (%)	Training cohort (%)	Testing cohort (%)	Measure calibration	Methods for handling missing values	External validation
Liu et al, 2019 [10]	China	Prospective cohort study	Cancer patients with PICCs^b^	PICC-related thrombosis	57/348 (16.38)	50	50	Monte Carlo cross-validation	No missing values	No
Nafee et al, 2020 [11]	America	Prospective follow-up study	Hospitalized medical illness (APEX^c^ trials)	VTE	405/7513 (5.39)	100	0	10-fold cross-validation	Exclude records with missing values	No
Wang et al, 2020 [12]	China	Retrospective study	Hospitalized patients	VTE	188/376	90	10	10-fold cross-validation	No missing values	No
Hou et al, 2021 [13]	China	Retrospective cohort study	Hospitalized patients	PE^d^	629/3619 (17.38)	80	20	5-fold cross-validation	Exclude records with missing values	No
Ryan et al,2021 [14]	America	Retrospective study	Hospitalized patients	DVT^e^	1230/99,412 (1.24)	80	20	5-fold cross-validation	Default path	No
Liu et al, 2021 [15]	China	Retrospective study	Young-middle-aged inpatients	VTE	167/573 (29.14)	75	25	10-fold cross-validation	KnnImputation method in the DMwR package	No
Ryan et al, 2022 [16]	America	Retrospective study	Hospitalized patient	PE	309/60,297 (0.51)	80	20	Cross‐validated grid search	Imputed missing values using the mean	No
Lei et al, 2022 [17]	China	Retrospective cohort study	Lung cancer patients	VTE	125/3398 (3.68)	64	20 testing and 16 validation	5-fold cross-validation	Imputed iteratively with the Bayesian ridge regression estimator	No
Jin et al, 2022 [8]	China	Retrospective cohort study	Patients with cancer	DVT	231/1035 (22.32)	70	30	10-fold cross-validation	Imputed missing values using the median	No
Yan et al, 2023 [18]	China	Retrospective, cross-sectional study	After inguinal hernia surgery	VTE	29/2856 (1.02)	80	20	Not reported	Random forest algorithm used for data-level imputation	Yes
Wang et al, 2023 [19]	China	Retrospective study	After knee/hip arthroplasty	DVT	1161/6897 (16.83)	80	20	5-fold cross-validation	No missing values	No
Wang et al, 2023 [20]	America	Retrospective study	After single-level posterior lumbar fusion	VTE	128/13,500(0.95)	80	20	Not reported	Excluded records with missing values	No
Shohat et al, 2023 [21]	America	Retrospective cohort study	Following total joint arthroplasty	VTE	308/35,963 (0.86)	70	30	Repeated cross-validation	No missing values	Yes
Sheng et al, 2023 [22]	China	Retrospective study	Hospital-wide patients at admission	VTE	263/3078 (8.52)	70	30	5-fold cross-validation	No missing values	No
Qin et al, 2023 [23]	China	Retrospective study	Colorectal cancer	VTE	129/1191 (10.83)	30	70	10-fold cross-validation	Multivariate imputation by chained equations method	No
Papillon et al, 2023 [24]	America	Retrospective study	Injured Children	VTE	521/347,576 (0.15)	66	34	5-fold cross-validation	Multiple Imputation	No
Ding et al, 2023 [25]	China	Retrospective study	Total hip arthroplasty	VTE	76/1481 (5.13)	70	30	10-fold cross-validation	Imputed missing values using the mean	No
Hou et al, 2023 [26]	China	Retrospective study	Stroke, traumatic brain injury, spinal cord injury patients	DVT	71/801 (8.86)	70	30	5-fold cross-validation	Imputed missing values using the mean and mode	No
Katiyar et al, 2023 [27]	America	Retrospective cohort study	After spine surgery	VTE	31/63 (49.21)	84	16	10-fold cross-validation	Imputation	No
Nassour et al, 2024 28]	China	Retrospective study	Ankle fractures	VTE	238/1175 (20.26)	60	20 testing and 20 validation	10-fold cross-validation	Performed only on the training set	No
Lin et al, 2024 [29]	China	Retrospective study	After hysterectomy among gynecological malignant tumor patients	VTE	65/1087 (5.98)	80	20	10-fold cross-validation	No missing values	No
Liu et al, 2024 [30]	China	Retrospective cohort study	After stroke	DVT	199/620 (32.1)	70	30	Not reported	Imputed missing values using random forest method	Yes
Wei et al, 2024 [31]	China	Retrospective study	After lower limb fracture surgery	DVT	207/4424 (4.68)	Not reported	Not reported	5-fold cross-validation	Multiple Imputation	No
Wu et al, 2024 [32]	China	Retrospective study	After spinal surgery	DVT	152/650 (23.39)	60	40	50 cross-validation	Imputed missing values using the mean	Yes
Zhou et al, 2024 [33]	China	Retrospective case‒control study	After radical gastrectomy for gastric cancer	DVT	38/693 (5.48)	70	30	Not reported	No missing values	No
Chen et al, 2024 [34]	China	Retrospective cohort study	After gynecological laparoscopy	DVT	41/489 (8.38)	70	30	Not reported	No missing values	No
Huang et al, 2024 [35]	China	Retrospective study	Hospitalized patients	PE	172/332	70	30	10-fold cross-validation	Imputed missing values using the mean	No

a VTE: venous thromboembolism.

b PICC: peripherally inserted central catheter.

c APEX: Acute Medically Ill VTE Prevention With Extended Duration Betrixaban.

d PE: pulmonary embolism.

e DVT: deep vein thrombosis.

### Results of the Quality Assessment

**Figure 2.**
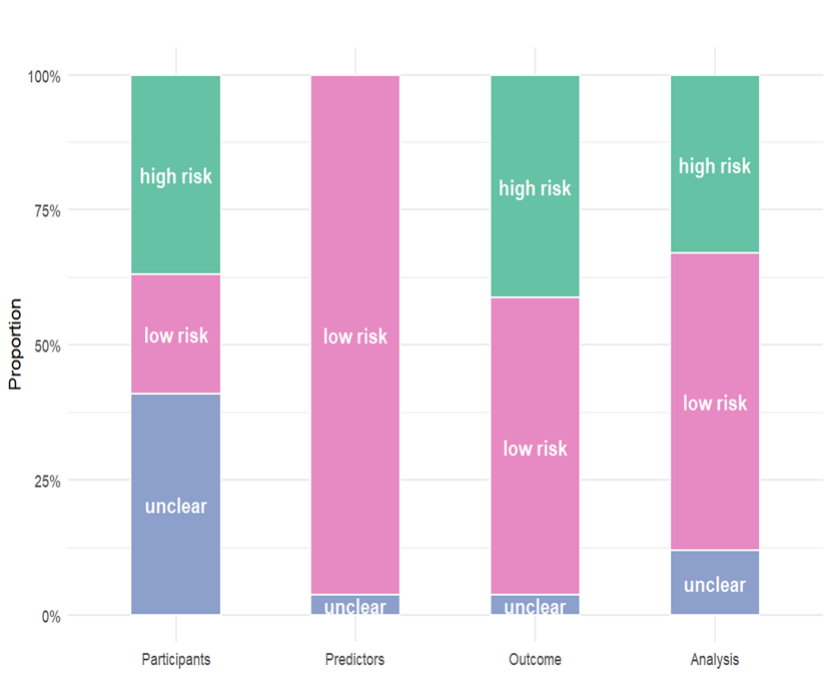
Summary of the risk of bias assessment for the 27 included studies on machine learning models for venous thromboembolism prediction using the Prediction Model Risk of Bias Assessment Tool. High, low, and unclear risks of bias are presented in green, pink, and blue, respectively.

A summary of the risk of bias assessment is shown in Figure 2. In the participant domain, among the 27 included studies, 6 (22%) had a low risk and 10 (37%) had a high risk of bias, primarily due to the study design, which was not a prospective, cross-sectional, or cohort study, and 11 (41%) did not report this. In the predictor domain, 26 (96%) studies demonstrated low risk, and 1 (4%) study had an unclear risk of bias because it did not report the quality control measures used for predictor assessment, possibly because of the retrospective design. In the outcome domain, 15 (56%) studies had a low risk and 11 (41%) studies were determined to have a high risk of bias, primarily due to failures in key methodological areas: 2 (18%) studies did not exclude predictors from the outcome definition, 6 (55%) studies did not report the consistency of the outcome definition and ascertainment, and 3 (27%) studies did not report the time interval between predictor assessment and outcome determination. A total of 1 (4%) additional study was rated as unclear risk due to insufficient reporting. Within the analysis domain, 15 (56%) studies had a low risk, 9 (33%) studies exhibited high-risk biases, with 6 (22%) studies inappropriately handling missing data or excluding enrolled participants, 2 (7%) studies neglecting to address model overfitting or underfitting, and 1 (4%) study not providing information regarding complexities in the data. Detailed information is provided in Multimedia Appendix 2.

### Meta-Analysis of Combined Effects

**Figure 3.**
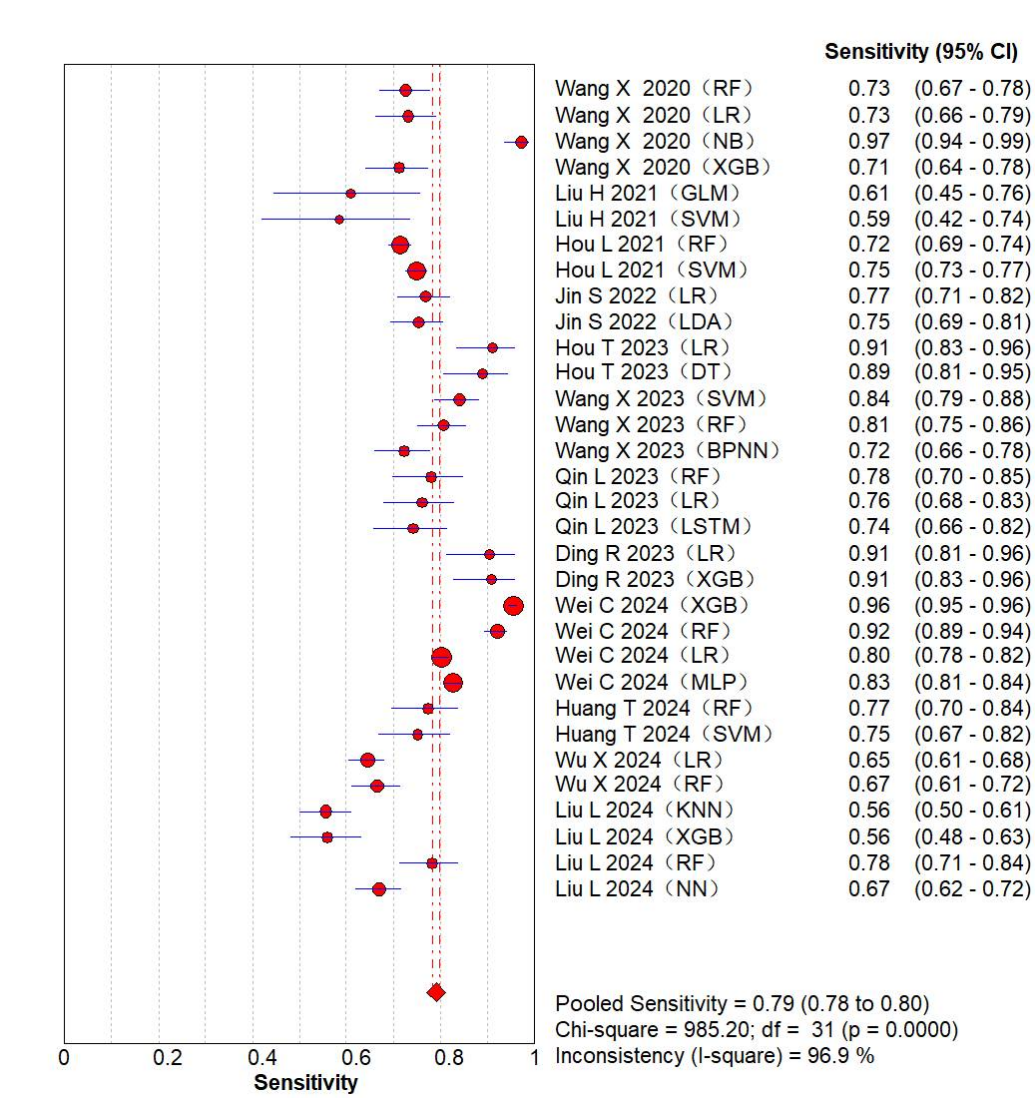
The overall pooled sensitivity of machine learning models for venous thromboembolism prediction. The first authors for each study are listed along the y-axis [8,12,13,15,19,23,25,26,30-32,35]. The vertical red dotted lines are the 95% CIs of the pooled sensitivity. BPNN: back propagation neural network; CT: classification tree; DT: decision tree; GBC: gradient boosting classification; GBDT: gradient boosting decision tree; GLM: generalized linear model; KNN: k-nearest neighbor; LR: logistic regression; LSTM: long short-term memory; MLP: multilayer perceptron; NB: naive Bayes; NN: neural network; RF: random forest; RPART: recursive partitioning and regression trees; SVM: support vector machine; XGB: extreme gradient boosting.

**Figure 4.**
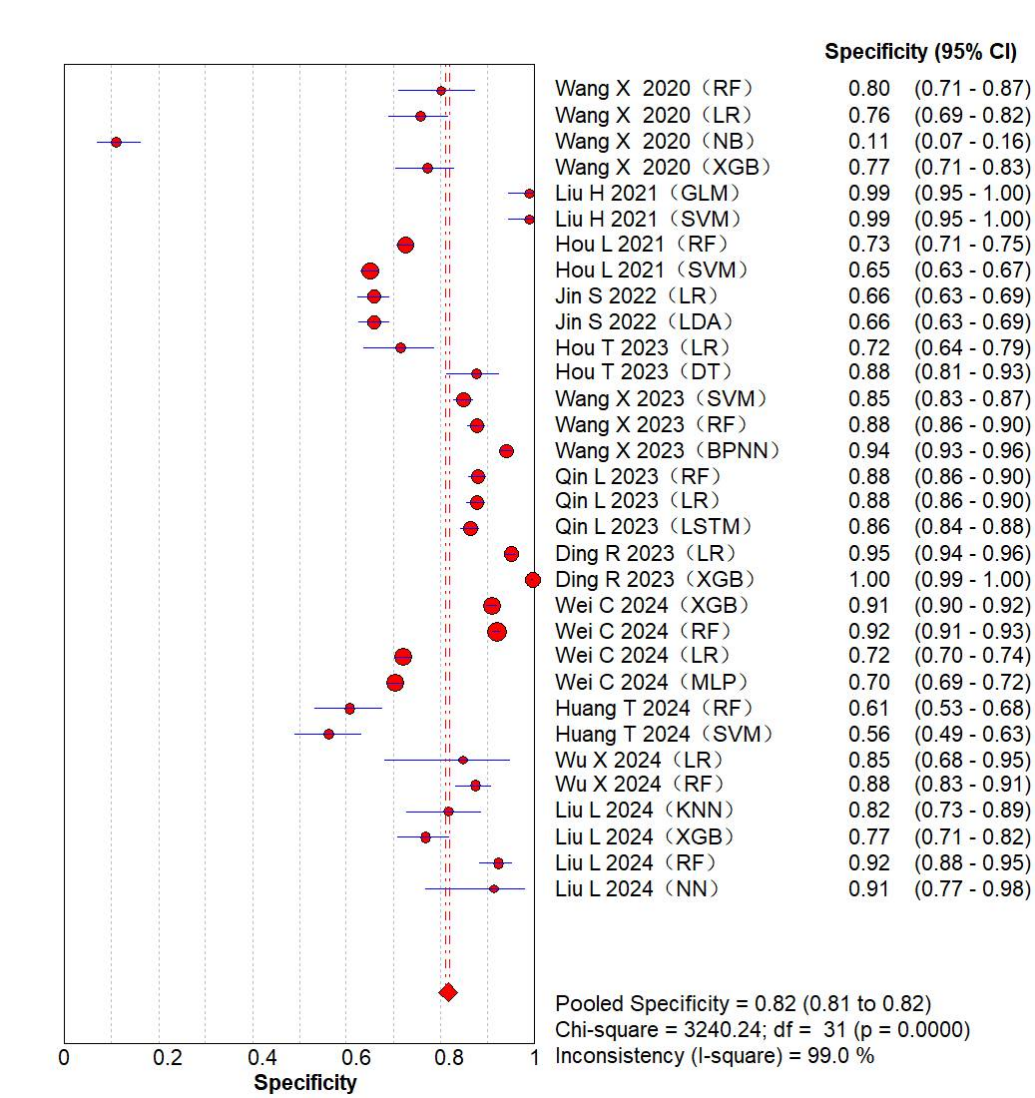
The overall pooled specificity of machine learning for venous thromboembolism prediction. The first authors for each study are listed along the y-axis [8,12,13,15,19,23,25,26,30-32,35]. The vertical red dotted line is the 95% CI of the pooled specificity. BPNN: back propagation neural network; CT: classification tree; DT: decision tree; GBC: gradient boosting classification; GBDT: gradient boosting decision tree; GLM: generalized linear model; KNN: k-nearest neighbor; LR: logistic regression; LSTM: long short-term memory; MLP: multilayer perceptron; NB: naive Bayes; NN: neural network; RF: random forest; RPART: recursive partitioning and regression trees; SVM: support vector machine; XGB: extreme gradient boosting.

The results of the meta-analysis for the 2 × 2 table showed additional values were as follows: sensitivity 0.79 (95% CI 0.78-0.80; *P*<.001; Figure 3 [8,12,13,15,19,23,25,26,30-32,35]) specificity 0.82 (95% CI 0.81-0.82; *P*<.001; Figure 4 [8,12,13,15,19,23,25,26,30-32,35]), positive likelihood ratio 5.02 (95% CI 3.81‐6.60; *P*<.001), negative likelihood ratio 0.27 (95% CI 0.22‐0.33; *P*<.001), and diagnostic odds ratio 20.14 (95% CI 13.69‐29.63; *P*<.001). A random-effects model was leveraged for meta-analysis of the C-index, which was 0.84 (95% CI 0.80‐0.88), as shown in Figure S1 in Multimedia Appendix 3.

### Publication Bias

To assess publication bias, we generated funnel plots using RStudio (RStudio, Inc) software. The visual inspection of the Deeks funnel plots suggested approximate symmetry (*P*=.067). This result indicates that the conclusions of this meta-analysis are robust and unaffected by the selective publication of studies (Figure S2 in Multimedia Appendix 3).

### Subgroup Analysis

We conducted subgroup analyses in 2 areas: venous thrombosis type (VTE, PE, and DVT) and ML algorithms (random forest, logistic regression, extreme gradient boosting, and support vector machine). To evaluate the performance of the predictive models built using different ML algorithms, subgroup analyses were performed for those models applied more than 4 times, as detailed in Table 2 and Figures S3-S6 in Multimedia Appendix 3. In the subgroup analysis of venous thrombosis types, DVT demonstrated the highest sensitivity (0.83, 95% CI 0.78-0.88), followed by VTE (0.82, 95% CI 0.70-0.94) and PE (0.76, 95% CI 0.72-0.80). Regarding specificity, the highest value was observed for VTE (0.91, 95% CI 0.81-0.99), followed by DVT (0.86, 95% CI 0.81-0.91) and PE (0.67, 95% CI 0.61-0.75). In the subgroup analysis of the ML algorithms, extreme gradient boosting achieved the highest estimates of both sensitivity and specificity.

**Table 2.** Subgroup analysis results for thrombosis types and machine learning algorithms.

Subgroup	Sensitivity (95% CI)	*P* value (sensitivity)	Specificity (95% CI)	*P* value (specificity)
Outcome				
VTE^a^	0.82 (0.70‐0.94)	<.001	0.91 (0.81‐0.99)	<.001
PE^b^	0.76 (0.72‐0.80)	.19	0.67 (0.61‐0.75)	.005
DVT^c^	0.83 (0.78‐0.88)	<.001	0.86 (0.81‐0.91)	.001
Machine learning				
RF^d^	0.77 (0.72‐0.82)	<.001	0.82 (0.78‐0.86)	<.001
LR^e^	0.78 (0.73‐0.83)	<.001	0.79 (0.74‐0.84)	<.001
XGB^f^	0.81 (0.75‐0.87)	<.001	0.88 (0.83‐0.92)	<.001
SVM^g^	0.72 (0.65‐0.79)	<.001	0.82 (0.79‐0.85)	<.001

a VTE: venous thromboembolism.

b PE: pulmonary embolism.

c DVT: deep vein thrombosis.

d RF: random forest.

e LR: logistic regression.

f XGB: extreme gradient boosting.

g SVM: support vector machine.

### Frequencies of the Use of Predictors for VTE

Regarding the use of predictors for VTE, there were a total of 55 predictors including age (n=12, 22%), D-dimer (n=7, 13%), VTE history (n=6, 11%), sex (n=3, 5%), ‌prothrombin time (n=3, 5%), chemotherapy (n=3, 5%), reduced mobility (n=2, 4%), cerebrovascular accident (n=2, 4%), blood type (n=2, 4%), active cancer (n=2, 4%), bedridden (n=2, 4%), BMI (n=2, 4%), varicose veins (n=2, 4%), prophylactic anticoagulants (n=2, 4%), hypoalbuminemia (n=2, 4%), international normalized ratio (n=2, 4%), fibrinogen level (n=2, 4%), and creatinine level (n=2, 4%). The following predictors were each used with a frequency of 1: thrombophiliac condition, heart or respiratory failure, activated partial thrombin time, infection or rheumatologic disorder, WBC, RBC, platelet count, Charlson Comorbidity Index, central venous catheter, Glasgow Coma Scale, C-reactive protein, epinephrine, Karnofsky performance status, Barthel index, mean corpuscular volume, Brunnstrom stage, Rocuronium consumption, anesthesia time, low-density lipoprotein, in-hospital duration, life-threatening illness, record of recent fracture, history of surgery, chronic bronchitis, oral contraceptives or hormone replacement, operative time, hematocrit, hypercoagulopathy, blood transfusion, anemia, direct bilirubin, indirect bilirubin, fibrinogen degradation products, trauma to treatment (days), smoking, diabetes, and atrial fibrillation. Given the substantial number of predictors, only predictors with a frequency greater than 1 were plotted on the pie chart (Multimedia Appendix 4).

## Discussion

### Principal Findings

The primary aim of this systematic review was to investigate the performance of ML models in predicting VTE. The pooled sensitivity, specificity, and C-index were 0.79 (95% CI 0.78‐0.80), 0.82 (95% CI 0.81-0.82), and 0.84 (95% CI 0.80-0.88), respectively, demonstrating robust predictive performance in the internal validation. Moreover, all the included studies were published within the last 6 years, with 66% (18/27) appearing in the past 2 years, indicating that ML will remain a key research focus in the foreseeable future.

Traditional scoring scales are usually based on physicians’ clinical experience and routine laboratory tests, which have certain subjective limitations. ML converts practical clinical problems into mathematical problems through computer simulation, classifies and processes big data, and screens and identifies high-risk populations. Previous studies have demonstrated that ML outperforms traditional statistical models when dealing with large datasets with many features. Among the included studies, 12 (44%) had sample sizes exceeding 1000, and 5 (19%) had sample sizes exceeding 10,000. On the basis of this, multiple types of data, such as laboratory indicators and metabolomics data, can be integrated using ML algorithms to improve the accuracy of VTE prediction and diagnosis. These large-scale studies validated the superior scalability and robustness of ML algorithms in handling high-dimensional clinical datasets.

### Predictor Selection and Clinical Applicability

In clinical practice, predictor selection is crucial for the performance and interpretation of models. Our study summarized the predictors with high frequencies. Age was the most frequently reported predictor, suggesting that advanced age is a high-risk factor for VTE. Prominent laboratory predictors, including D-dimer, prothrombin time, fibrinogen, and activated partial thromboplastin time levels, are critical for evaluating coagulation abnormalities. Interestingly, nonlaboratory predictors, such as chemotherapy, reduced mobility, and active cancer, have also been reported, reflecting the multifactorial nature of VTE risk. These findings align with the existing literature that integrates laboratory and clinical data for comprehensive VTE risk stratification. The diversity of these factors reflects the complexity of VTE diagnosis, necessitating a comprehensive evaluation tailored to the individual patient profile.

The reporting of high-risk factors was inconsistent across the included studies, which appears to be primarily attributable to the geographic and ethnic differences in study populations, which may further influence risk factor profiles, as genetic predispositions and environmental exposures vary across regions. Furthermore, the heterogeneous definitions of *reduced mobility* across studies may lead to inconsistent identification of its association with VTE risk, potentially obscuring its true clinical significance as a predictor. Notably, less frequently cited predictors (thrombophilia, heart or respiratory failure, and inflammatory markers) appeared in single studies, suggesting that their associations may be population specific or may require further validation in larger studies with more robust datasets. Future studies should prioritize the standardized reporting of risk factors and validate their predictive utility across diverse populations to refine prediction models.

Over the past few years, several studies have attempted to predict VTE using genomic technologies. He et al [36] performed an exome-wide association study of VTE among 14,723 cases and 334,315 controls and found that protein C, protein pS1, serpin family C member 1, protein C receptor, coagulation factor V, Kallikrein B1, fibrinogen alpha chain, stabilin 2, von Willebrand factor, serine/arginine–rich splicing factor 6, phosphohistidine phosphatase 1, cingulin, and mitogen-activated protein kinase 2 were associated with VTE risk. Another meta-analysis showed that the variants G20210A (c.*97G>A) in F2 and p.R534Q in F5 were linked to the risk of VTE [37]. Although genomic technology has demonstrated some value and significance in the prediction of VTE, considering the higher cost and time-consuming nature of genomic technologies, as well as the complexity of genomic data analysis and interpretation, genomic technology is less clinically applicable to the prediction of VTE. Further research is warranted to validate the predictive value of these factors, particularly in high-risk populations, and to explore how these factors interact in predictive models to optimize individualized prophylaxis.

Furthermore, thromboelastography is an important predictor of VTE. A meta-analysis by Brown et al [38] showed that the maximum amplitude value was associated with the risk of postoperative VTE. However, there remains broad variability in the definition of hypercoagulability as determined by the maximum amplitude, which limits its predictive ability and results in less application. In contrast, the common clinical variables described in this report are more applicable and convenient in most current clinical settings while being more conducive to reducing the economic burden and time cost for people with VTE.

### Limitations and Future Directions

However, ML models have certain limitations. Their current clinical utility is limited by the high risk of bias, heterogeneity, and lack of external validation in the underlying studies. Therefore, these results should be interpreted with caution.

Although the results indicated favorable predictive performance, the high risk of bias identified in 18 (67%) of the 27 studies using the PROBAST assessment substantially limited the clinical applicability and generalizability of these models. Three major bias domains account for these limitations. First, in terms of study design, of the 27 included studies, only 2 (7%) were prospective studies. This heavy reliance on retrospective data increases the susceptibility to selection bias and unmeasured confounding factors. This methodological gap underscores the need for well-designed prospective studies to reduce heterogeneity in future research. Second, the methods for handling missing data introduced potential biases. Among the included studies, 8 (30%) reported complete data with no missing values, thus avoiding potential biases. However, 3 (11%) studies excluded records with missing values and introduced selection bias. Another 5 (19%) studies used simple imputation, such as mean substitution, which distorts the correlations between variables and may introduce bias by underestimating SEs. More robust methods were also used: 1 (4%) study applied k-nearest neighbor imputation to preserve local relationships, and another used Bayesian ridge regression to account for uncertainty. Additionally, 2 (7%) studies used random forest imputation for nonlinear patterns, and 2 (7%) implemented multiple imputations to capture variability. Future research should prioritize transparent reporting and advanced methods, such as multiple imputation, to minimize bias. Third, only 4 (15%) of the 27 included studies performed external validation, which may have limited the generalizability of the prediction models to broader populations. This lack of external validation raises concerns about potential overfitting and may overestimate model performance when applied in real clinical settings. To mitigate this, future research should prioritize multicenter collaborations to establish diverse validation cohorts that reflect real clinical heterogeneity.

The interpretation of our findings is constrained by the significant heterogeneity stemming from multiple aspects of the included studies. First, the included studies used diverse ML models, each with distinct operational mechanisms. Random forest uses ensemble learning, aggregating predictions from multiple decision trees via bagging and random feature selection, thereby enhancing accuracy through majority voting. In contrast, support vector machines optimize classification using kernel functions and demonstrate strong performance in medium-sized datasets. Such methodological differences may contribute to heterogeneity in model performance and generalizability. Second, this study included diverse populations, such as hospitalized medical patients, patients with cancers, postoperative groups (eg, patients who underwent arthroplasty and spinal surgery), and individuals with specific conditions (eg, stroke or trauma). The observed heterogeneity likely arises from variations in clinical contexts and baseline risk profiles across patients. For instance, medically ill inpatients and surgical patients differ substantially in terms of thrombotic risk and comorbidities, which may contribute to differences in the model performance. Third, the heterogeneity in our findings may be partially attributed to variations in the target VTE outcomes across studies, which included PE, DVT, and peripherally inserted central catheter–related VTE. These thrombotic manifestations differ substantially in pathophysiology and diagnostic criteria. Fourth, the heterogeneity observed across the studies may be attributed to the varying strategies used for dataset partitioning. Although 16 (59%) of the 27 studies used conventional splits (a ratio of 70:30 or 80:20 for training and testing), we discovered different approaches, including an unbalanced 30:70 ratio for training and testing and the implementation of triple-split designs with dedicated validation sets. This methodological variation highlights the need for standardized data splitting to ensure a fair comparison. Finally, the Deeks funnel plot indicated potential heterogeneity, with smaller studies (effective sample size <200) showing greater dispersion and possible bias, while larger studies (effective sample size >400) clustered more tightly around the mean effect. This asymmetry suggests that the variability in smaller studies may disproportionately influence the overall results. Thus, future studies should be prospective with larger sample sizes, adhere to standardized reporting protocols, and maintain complete transparency in methodology reporting, thereby enhancing the generalizability of VTE predictive models.

### Ethical Issues

Although our review indicates that ML models can achieve relatively satisfactory accuracy for VTE prediction, several ethical issues inherent to ML in medicine should be acknowledged, including data bias, lack of transparency, the black box phenomenon, issues regarding data privacy, and responsibility for decision-making [39]. The algorithmic bias of ML poses a significant threat to health equity. As models learn from human-generated data, which often contain inherent biases, these biases are inevitable [40]. If trained on nonrepresentative datasets, these models have the potential to perpetuate or even amplify existing biases, resulting in compromised predictive performance and unjust outcomes in underrepresented patient populations. Therefore, future developments must prioritize the use of diverse multicenter datasets and use rigorous fairness audits to identify and mitigate these biases. Autonomous decision-making by clinicians and patients requires an understanding of the process. The “black box” (or “brain”) nature of many complex ML models erodes this autonomy [41]. The lack of transparency and intuitive explanations for predictions complicates clinical validation and hinders the detection of model errors and biases. Furthermore, the development of these models relies on large volumes of sensitive data. Inadequate data protection risks privacy breaches, potentially causing psychological, social, and economic harm.

To comprehensively analyze the ethical considerations of deploying ML in VTE prediction, we framed our discussion using the widely accepted framework of biomedical ethics [42], which includes respect for autonomy, beneficence, nonmaleficence, and justice. First, the principle of justice demands proactive auditing of algorithmic bias to ensure equitable model performance across all demographic groups, thus preventing the exacerbation of health care disparities. Second, beneficence and nonmaleficence mandate that models be not only accurate but also transparent. Consequently, integrating explainable artificial intelligence techniques, such as Shapley additive explanations, is crucial for demystifying the black box nature of complex models. This enhances transparency and enables clinicians to understand and trust the VTE prediction outputs. However, our review discovered that only 6 (22%) of the 27 studies used the Shapley additive explanations technique to interpret their model predictions. This scarcity highlights a significant limitation of model interpretability in the existing literature (Multimedia Appendix 5), underscoring the imperative for future research to prioritize the integration of explainable prediction models. Finally, given that these systems rely on large volumes of sensitive patient data, their reliance on large volumes of data necessitates robust privacy protection protocols.

### Conclusions

The use of ML-based models for predicting VTE is increasing and may potentially improve existing models. In conclusion, ML has a favorable predictive performance for venous thrombosis risk and can be used as a potential tool for the early identification of venous thrombosis risk. However, the development of ML models in related fields is still limited by the lack of external validation and a high risk of bias, which reduces their practical application in clinical practice. Future research should prioritize the development of prediction models with larger sample sizes, rigorous research designs, external validation, and scientific reports to enhance the practical clinical application capabilities of these models.

## Supplementary material

10.2196/77339 Multimedia Appendix 1 Search strategy.

10.2196/77339 Multimedia Appendix 2 Risk of bias assessment.

10.2196/77339 Multimedia Appendix 3 Meta-analysis.

10.2196/77339 Multimedia Appendix 4 Frequency of predictors inclusion in machine learning models for venous thromboembolism based on the included studies.

10.2196/77339 Multimedia Appendix 5 Use of Shapley Additive Explanation for model interpretation.

10.2196/77339 Checklist 1 PRISMA checklist.

## Abbreviations

CHARMS: Critical Appraisal and Data Extraction for Systematic Reviews of Prediction Modeling Studies
DVT: deep vein thrombosis
FN: false negative
FP: false positive
ML: machine learning
PE: pulmonary embolism
PRISMA: Preferred Reporting Items for Systematic Reviews and Meta-Analyses
PROBAST: Prediction Model Risk of Bias Assessment Tool
PROSPERO: International Prospective Register of Systematic Reviews
TN: true negative
TP: true positive
VTE: venous thromboembolism
