# Supplementary material for: Machine Learning in the Prediction of Venous Thromboembolism: Systematic Review and Meta-Analysis
Source: J Med Internet Res. 2025 Dec 23;27:e77339. doi: 10.2196/77339 (PMC12724482; doi:10.2196/77339)
Supplement: Multimedia Appendix 1 [file jmir-v27-e77339-s001.doc]

**1. PubM**ed

| Search number | Query | Results |
| --- | --- | --- |
| 3 | ("Machine Learning"[Mesh Terms] OR "learning, machine"[Title/Abstract] OR "transfer learning"[Title/Abstract] OR "learning, transfer"[Title/Abstract]) AND (((("Venous Thromboembolism"[Mesh]) OR ("Venous Thrombosis"[Mesh])) OR ("Pulmonary Embolism"[Mesh])) OR (Thromboembolism, Venous[Title/Abstract] OR Thromboses, Venous[Title/Abstract] OR Venous Thromboses[Title/Abstract] OR Deep Vein Thrombosis[Title/Abstract] OR Deep Vein Thromboses[Title/Abstract] OR Phlebothrombosis[Title/Abstract] OR Phlebothromboses[Title/Abstract] OR Thrombosis, Venous[Title/Abstract] OR Thromboses, Deep Vein[Title/Abstract] OR Vein Thromboses, Deep[Title/Abstract] OR Vein Thrombosis, Deep[Title/Abstract] OR Deep-Venous Thrombosis[Title/Abstract] OR Deep-Venous Thromboses[Title/Abstract] OR Thromboses, Deep-Venous[Title/Abstract] OR Thrombosis, Deep-Venous[Title/Abstract] OR Deep-Vein Thrombosis[Title/Abstract] OR Deep-Vein Thromboses[Title/Abstract] OR Thromboses, Deep-Vein[Title/Abstract] OR Thrombosis, Deep-Vein[Title/Abstract] OR Thrombosis, Deep Vein[Title/Abstract] OR Deep Venous Thrombosis[Title/Abstract] OR Deep Venous Thromboses[Title/Abstract] OR Thromboses, Deep Venous[Title/Abstract] OR Thrombosis, Deep Venous[Title/Abstract] OR Venous Thromboses, Deep[Title/Abstract] OR Venous Thrombosis, Deep[Title/Abstract] OR Pulmonary Embolisms[Title/Abstract] OR Embolism, Pulmonary[Title/Abstract] OR Embolisms, Pulmonary[Title/Abstract] OR Pulmonary Thromboembolisms[Title/Abstract] OR Pulmonary Thromboembolism[Title/Abstract] OR Thromboembolism, Pulmonary[Title/Abstract] OR Thromboembolisms, Pulmonary[Title/Abstract])) | 191 |
| 2 | ((("Venous Thromboembolism"[Mesh]) OR ("Venous Thrombosis"[Mesh])) OR ("Pulmonary Embolism"[Mesh])) OR (Thromboembolism, Venous[Title/Abstract] OR Thromboses, Venous[Title/Abstract] OR Venous Thromboses[Title/Abstract] OR Deep Vein Thrombosis[Title/Abstract] OR Deep Vein Thromboses[Title/Abstract] OR Phlebothrombosis[Title/Abstract] OR Phlebothromboses [Title/Abstract] OR Thrombosis, Venous[Title/Abstract] OR Thromboses, Deep Vein[Title/Abstract] OR Vein Thromboses, Deep[Title/Abstract] OR Vein Thrombosis, Deep[Title/Abstract] OR Deep-Venous Thrombosis[Title/Abstract] OR Deep-Venous Thromboses[Title/Abstract] OR Thromboses, Deep-Venous[Title/Abstract] OR Thrombosis, Deep-Venous[Title/Abstract] OR Deep-Vein Thrombosis[Title/Abstract] OR Deep-Vein Thromboses[Title/Abstract] OR Thromboses, Deep-Vein[Title/Abstract] OR Thrombosis, Deep-Vein[Title/Abstract] OR Thrombosis, Deep Vein[Title/Abstract] OR Deep Venous Thrombosis[Title/Abstract] OR Deep Venous Thromboses[Title/Abstract] OR Thromboses, Deep Venous[Title/Abstract] OR Thrombosis, Deep Venous[Title/Abstract] OR Venous Thromboses, Deep[Title/Abstract] OR Venous Thrombosis, Deep[Title/Abstract] OR Pulmonary Embolisms[Title/Abstract] OR Embolism, Pulmonary[Title/Abstract] OR Embolisms, Pulmonary[Title/Abstract] OR Pulmonary Thromboembolisms[Title/Abstract] OR Pulmonary Thromboembolism[Title/Abstract] OR Thromboembolism, Pulmonary[Title/Abstract] OR Thromboembolisms, Pulmonary[Title/Abstract]) | 128518 |
| 1 | "Machine Learning"[Mesh Terms] OR "learning, machine"[Title/Abstract] OR "transfer learning"[Title/Abstract] OR "learning, transfer"[Title/Abstract] | 95081 |

**2. Web of science**

| Search number | Search Query | Results |
| --- | --- | --- |
| 1 | (TI=(Machine Learning OR learning, machine OR transfer learning OR learning, transfer)) OR AB=(Machine Learning OR learning, machine OR transfer learning OR learning, transfer) | 524569 |
| 2 | (TI=(Venous Thromboembolism OR Venous Thrombosis OR Pulmonary Embolism OR Thromboembolism, Venous OR Thromboses, Venous OR Venous Thromboses OR Deep Vein Thrombosis OR Deep Vein Thromboses OR Phlebothrombosis OR Phlebothromboses OR Thrombosis, Venous OR Thromboses, Deep Vein OR Vein Thromboses, Deep OR Vein Thrombosis, Deep OR Deep-Venous Thrombosis OR Deep-Venous Thromboses OR Thromboses, Deep-Venous OR Thrombosis, Deep-Venous OR Deep-Vein Thrombosis OR Deep-Vein Thromboses OR Thromboses, Deep-Vein OR Thrombosis, Deep-Vein OR Thrombosis, Deep Vein OR Deep Venous Thrombosis OR Deep Venous Thromboses OR Thromboses, Deep Venous OR Thrombosis, Deep Venous OR Venous Thromboses, Deep OR Venous Thrombosis, Deep OR Pulmonary Embolisms OR Embolism, Pulmonary OR Embolisms, Pulmonary OR Pulmonary Thromboembolisms OR Pulmonary Thromboembolism OR Thromboembolism, Pulmonary OR Thromboembolisms, Pulmonary)) OR AB=(Venous Thromboembolism OR Venous Thrombosis OR Pulmonary Embolism OR Thromboembolism, Venous OR Thromboses, Venous OR Venous Thromboses OR Deep Vein Thrombosis OR Deep Vein Thromboses OR Phlebothrombosis OR Phlebothromboses OR Thrombosis, Venous OR Thromboses, Deep Vein OR Vein Thromboses, Deep OR Vein Thrombosis, Deep OR Deep-Venous Thrombosis OR Deep-Venous Thromboses OR Thromboses, Deep-Venous OR Thrombosis, Deep-Venous OR Deep-Vein Thrombosis OR Deep-Vein Thromboses OR Thromboses, Deep-Vein OR Thrombosis, Deep-Vein OR Thrombosis, Deep Vein OR Deep Venous Thrombosis OR Deep Venous Thromboses OR Thromboses, Deep Venous OR Thrombosis, Deep Venous OR Venous Thromboses, Deep OR Venous Thrombosis, Deep OR Pulmonary Embolisms OR Embolism, Pulmonary OR Embolisms, Pulmonary OR Pulmonary Thromboembolisms OR Pulmonary Thromboembolism OR Thromboembolism, Pulmonary OR Thromboembolisms, Pulmonary) | 97603 |
| 3 | #1 AND #2 | 346 |

**3.** [**MEDLINE**](https://www.baidu.com/link?url=7EtJ1tCobopBloktrkSZ4U-U5Ok4vJF3zsUSxK-IYE8bwQ7XK0a6kxZ0gWVCUjcUg6vVzcxIBkP3XNeTsA1EvyD1iQt8kRrTLSKRKLY-nDG&wd=&eqid=a342239b00610c440000000265e82cda)

| Search number | Query | Results |
| --- | --- | --- |
| 1 | (TI=(Machine Learning OR learning, machine OR transfer learning OR learning, transfer)) OR AB=(Machine Learning OR learning, machine OR transfer learning OR learning, transfer) Editions: MEDLINE.MEDLINE | 151510 |
| 2 | (TI=(Venous Thromboembolism OR Venous Thrombosis OR Pulmonary Embolism OR Thromboembolism, Venous OR Thromboses, Venous OR Venous Thromboses OR Deep Vein Thrombosis OR Deep Vein Thromboses OR Phlebothrombosis OR Phlebothromboses OR Thrombosis, Venous OR Thromboses, Deep Vein OR Vein Thromboses, Deep OR Vein Thrombosis, Deep OR Deep-Venous Thrombosis OR Deep-Venous Thromboses OR Thromboses, Deep-Venous OR Thrombosis, Deep-Venous OR Deep-Vein Thrombosis OR Deep-Vein Thromboses OR Thromboses, Deep-Vein OR Thrombosis, Deep-Vein OR Thrombosis, Deep Vein OR Deep Venous Thrombosis OR Deep Venous Thromboses OR Thromboses, Deep Venous OR Thrombosis, Deep Venous OR Venous Thromboses, Deep OR Venous Thrombosis, Deep OR Pulmonary Embolisms OR Embolism, Pulmonary OR Embolisms, Pulmonary OR Pulmonary Thromboembolisms OR Pulmonary Thromboembolism OR Thromboembolism, Pulmonary OR Thromboembolisms, Pulmonary)) OR AB=(Venous Thromboembolism OR Venous Thrombosis OR Pulmonary Embolism OR Thromboembolism, Venous OR Thromboses, Venous OR Venous Thromboses OR Deep Vein Thrombosis OR Deep Vein Thromboses OR Phlebothrombosis OR Phlebothromboses OR Thrombosis, Venous OR Thromboses, Deep Vein OR Vein Thromboses, Deep OR Vein Thrombosis, Deep OR Deep-Venous Thrombosis OR Deep-Venous Thromboses OR Thromboses, Deep-Venous OR Thrombosis, Deep-Venous OR Deep-Vein Thrombosis OR Deep-Vein Thromboses OR Thromboses, Deep-Vein OR Thrombosis, Deep-Vein OR Thrombosis, Deep Vein OR Deep Venous Thrombosis OR Deep Venous Thromboses OR Thromboses, Deep Venous OR Thrombosis, Deep Venous OR Venous Thromboses, Deep OR Venous Thrombosis, Deep OR Pulmonary Embolisms OR Embolism, Pulmonary OR Embolisms, Pulmonary OR Pulmonary Thromboembolisms OR Pulmonary Thromboembolism OR Thromboembolism, Pulmonary OR Thromboembolisms, Pulmonary) Editions: MEDLINE.MEDLINE | 122142 |
| 3 | #1 AND #2 Editions: MEDLINE.MEDLINE | 312 |

**4.Embase**

| Search number | Query | Results |
| --- | --- | --- |
| #13 | #3 AND #12 | 1718 |
| #12 | #4 OR #5 OR #6 OR #7 OR #8 OR #9 OR #10 OR #11 | 333611 |
| #11 | 'acute deep venous thrombosis'/exp OR 'acute deep venous thrombosis' OR 'acute dvt'/exp OR 'acute dvt' OR 'deep thrombo-phlebitis'/exp OR 'deep thrombo-phlebitis' OR 'deep thrombophlebitis'/exp OR 'deep thrombophlebitis' OR 'deep vein blood clots'/exp OR 'deep vein blood clots' OR 'deep vein thrombophlebitis'/exp OR 'deep vein thrombophlebitis' OR 'deep vein thrombus'/exp OR 'deep vein thrombus' OR 'deep venous thrombophlebitis'/exp OR 'deep venous thrombophlebitis' OR 'deep venous thrombosis'/exp OR 'deep venous thrombosis' OR 'deep venous thrombus'/exp OR 'deep venous thrombus' OR 'dvt (deep vein thrombosis)'/exp OR 'dvt (deep vein thrombosis)' OR 'recurrent dvt'/exp OR 'recurrent dvt' OR 'thrombosis, acute deep venous'/exp OR 'thrombosis, acute deep venous' OR 'deep vein thrombosis'/exp OR 'deep vein thrombosis' | 103772 |
| #10 | 'deep vein thrombosis'/exp AND [embase]/lim | 90464 |
| #9 | 'chronic lung embolism'/exp OR 'chronic lung embolism' OR 'embolism, lung'/exp OR 'embolism, lung' OR 'lung embolization'/exp OR 'lung embolization' OR 'lung embolus'/exp OR 'lung embolus' OR 'lung embolus recurrence'/exp OR 'lung embolus recurrence' OR 'lung emboly'/exp OR 'lung emboly' OR 'lung microembolism'/exp OR 'lung microembolism' OR 'lung microembolization'/exp OR 'lung microembolization' OR 'lung microembolus'/exp OR 'lung microembolus' OR 'lung thromboembolism'/exp OR 'lung thromboembolism' OR 'microembolus, lung'/exp OR 'microembolus, lung' OR 'pulmonary embolism'/exp OR 'pulmonary embolism' OR 'pulmonary embolization'/exp OR 'pulmonary embolization' OR 'pulmonary embolus'/exp OR 'pulmonary embolus' OR 'pulmonary microembolism'/exp OR 'pulmonary microembolism' OR 'pulmonary thromboembolic disease'/exp OR 'pulmonary thromboembolic disease' OR 'pulmonary thromboembolism'/exp OR 'pulmonary thromboembolism' OR 'thromboembolism, lung'/exp OR 'thromboembolism, lung' OR 'lung embolism'/exp OR 'lung embolism' | 150316 |
| #8 | 'lung embolism'/exp AND [embase]/lim | 124356 |
| #7 | 'thromboembolism, venous'/exp OR 'thromboembolism, venous' OR 'vein thromboembolism'/exp OR 'vein thromboembolism' OR 'venous thromboembolism'/exp OR 'venous thromboembolism' | 234134 |
| #6 | 'venous thromboembolism'/exp AND [embase]/lim | 211421 |
| #5 | 'phlebo-thrombosis'/exp OR 'phlebo-thrombosis' OR 'phlebothrombosis'/exp OR 'phlebothrombosis' OR 'thrombosis, venous'/exp OR 'thrombosis, venous' OR 'vena thrombosis'/exp OR 'vena thrombosis' OR 'venothrombosis'/exp OR 'venothrombosis' OR 'venothrombotic event'/exp OR 'venothrombotic event' OR 'venous thrombosis'/exp OR 'venous thrombosis' OR 'vein thrombosis'/exp OR 'vein thrombosis' | 201526 |
| #4 | 'vein thrombosis'/exp AND [embase]/lim | 164950 |
| #3 | #1 OR #2 | 589579 |
| #2 | 'learning machine'/exp OR 'learning machine' OR 'learning machines'/exp OR 'learning machines' OR 'machine learning'/exp OR 'machine learning' | 589579 |
| #1 | ('machine learning'/exp OR 'machine learning') AND [embase]/lim | 456198 |

**5**.Cochrane

| search number | Query | Results |
| --- | --- | --- |
| #1 | MeSH descriptor: [Machine Learning] explode all trees | 1091 |
| #2 | (Transfer Learning; Learning, Transfer; Learning, Machine):ti,ab,kw (Word variations have been searched) | 5604 |
| #3 | #1 or #2 | 5938 |
| #4 | MeSH descriptor: [Venous Thromboembolism] explode all trees | 1316 |
| #5 | (Thromboembolism, Venous):ti,ab,kw (Word variations have been searched) | 6124 |
| #6 | MeSH descriptor: [Venous Thrombosis] explode all trees | 3526 |
| #7 | (Thrombosis, Deep-Vein; Deep-Vein Thromboses; Thromboses, Deep Venous; Thrombosis, Deep Venous; Vein Thromboses, Deep; Thromboses, Deep-Vein; Venous Thromboses, Deep; Deep Venous Thromboses; Vein Thrombosis, Deep; Deep Venous Thrombosis; Thromboses, Deep Vein; Thromboses, Deep-Venous; Thrombosis, Deep-Venous; Deep-Venous Thrombosis; Deep Vein Thrombosis; Deep Vein Thromboses; Deep-Vein Thrombosis; Venous Thrombosis, Deep; Deep-Venous Thromboses; Thrombosis, Deep Vein; Thromboses, Venous; Phlebothromboses; Venous Thromboses; Thrombosis, Venous; Phlebothrombosis):ti,ab,kw (Word variations have been searched) | 10406 |
| #8 | MeSH descriptor: [Pulmonary Embolism] explode all trees | 1519 |
| #9 | (Pulmonary Embolisms; Embolisms, Pulmonary; Embolism, Pulmonary; Pulmonary Thromboembolisms; Pulmonary Thromboembolism; Thromboembolisms, Pulmonary; Thromboembolism, Pulmonary):ti,ab,kw (Word variations have been searched) | 5616 |
| #10 | #4 or #5 or #6 or #7 or #8 #9 | 17121 |
| #11 | #3 and #10 | 29 |

**6.CINAHL**

| Search number | Query | Results |
| --- | --- | --- |
| S6 | (MH (MH "Venous Thromboembolism") OR TI Venous Thromboembolism OR Venous Thrombosis OR Pulmonary Embolism OR Thromboembolism, Venous OR Thromboses, Venous OR Venous Thromboses OR Deep Vein Thrombosis OR Deep Vein Thromboses OR Phlebothrombosis OR Phlebothromboses OR Thrombosis, Venous OR Thromboses, Deep Vein OR Vein Thromboses, Deep OR Vein Thrombosis, Deep OR Deep-Venous Thrombosis OR Deep-Venous Thromboses OR Thromboses, Deep-Venous OR Thrombosis, Deep-Venous OR Deep-Vein Thrombosis OR Deep-Vein Thromboses OR Thromboses, Deep-Vein OR Thrombosis, Deep-Vein OR Thrombosis, Deep Vein OR Deep Venous Thrombosis OR Deep Venous Thromboses OR Thromboses, Deep Venous OR Thrombosis, Deep Venous OR Venous Thromboses, Deep OR Venous Thrombosis, Deep OR Pulmonary Embolisms OR Embolism, Pulmonary OR Embolisms, Pulmonary OR Pulmonary Thromboembolisms OR Pulmonary Thromboembolism OR Thromboembolism, Pulmonary OR Thromboembolisms, Pulmonary OR AB Venous Thromboembolism OR Venous Thrombosis OR Pulmonary Embolism OR Thromboembolism, Venous OR Thromboses, Venous OR Venous Thromboses OR Deep Vein Thrombosis OR Deep Vein Thromboses OR Phlebothrombosis OR Phlebothromboses OR Thrombosis, Venous OR Thromboses, Deep Vein OR Vein Thromboses, Deep OR Vein Thrombosis, Deep OR Deep-Venous Thrombosis OR Deep-Venous Thromboses OR Thromboses, Deep-Venous OR Thrombosis, Deep-Venous OR Deep-Vein Thrombosis OR Deep-Vein Thromboses OR Thromboses, Deep-Vein OR Thrombosis, Deep-Vein OR Thrombosis, Deep Vein OR Deep Venous Thrombosis OR Deep Venous Thromboses OR Thromboses, Deep Venous OR Thrombosis, Deep Venous OR Venous Thromboses, Deep OR Venous Thrombosis, Deep OR Pulmonary Embolisms OR Embolism, Pulmonary OR Embolisms, Pulmonary OR Pulmonary Thromboembolisms OR Pulmonary Thromboembolism OR Thromboembolism, Pulmonary OR Thromboembolisms, Pulmonary) AND (S2 AND S5) | 28 |
| S5 | MH (MH "Venous Thromboembolism") OR TI ( Venous Thromboembolism OR Venous Thrombosis OR Pulmonary Embolism OR Thromboembolism, Venous OR Thromboses, Venous OR Venous Thromboses OR Deep Vein Thrombosis OR Deep Vein Thromboses OR Phlebothrombosis OR Phlebothromboses OR Thrombosis, Venous OR Thromboses, Deep Vein OR Vein Thromboses, Deep OR Vein Thrombosis, Deep OR Deep-Venous Thrombosis OR Deep-Venous Thromboses OR Thromboses, Deep-Venous OR Thrombosis, Deep-Venous OR Deep-Vein Thrombosis OR Deep-Vein Thromboses OR Thromboses, Deep-Vein OR Thrombosis, Deep-Vein OR Thrombosis, Deep Vein OR Deep Venous Thrombosis OR Deep Venous Thromboses OR Thromboses, Deep Venous OR Thrombosis, Deep Venous OR Venous Thromboses, Deep OR Venous Thrombosis, Deep OR Pulmonary Embolisms OR Embolism, Pulmonary OR Embolisms, Pulmonary OR Pulmonary Thromboembolisms OR Pulmonary Thromboembolism OR Thromboembolism, Pulmonary OR Thromboembolisms, Pulmonary ) OR AB ( Venous Thromboembolism OR Venous Thrombosis OR Pulmonary Embolism OR Thromboembolism, Venous OR Thromboses, Venous OR Venous Thromboses OR Deep Vein Thrombosis OR Deep Vein Thromboses OR Phlebothrombosis OR Phlebothromboses OR Thrombosis, Venous OR Thromboses, Deep Vein OR Vein Thromboses, Deep OR Vein Thrombosis, Deep OR Deep-Venous Thrombosis OR Deep-Venous Thromboses OR Thromboses, Deep-Venous OR Thrombosis, Deep-Venous OR Deep-Vein Thrombosis OR Deep-Vein Thromboses OR Thromboses, Deep-Vein OR Thrombosis, Deep-Vein OR Thrombosis, Deep Vein OR Deep Venous Thrombosis OR Deep Venous Thromboses OR Thromboses, Deep Venous OR Thrombosis, Deep Venous OR Venous Thromboses, Deep OR Venous Thrombosis, Deep OR Pulmonary Embolisms OR Embolism, Pulmonary OR Embolisms, Pulmonary OR Pulmonary Thromboembolisms OR Pulmonary Thromboembolism OR Thromboembolism, Pulmonary OR Thromboembolisms, Pulmonary ) | 30222 |
| S4 | (MH "Venous Thromboembolism") | 7948 |
| S3 | (MH "Venous Thrombosis") | 11270 |
| S2 | MH (MH "Machine Learning") OR TI ( Learning, Machine OR Transfer Learning OR Learning, Transfer ) OR AB ( Learning, Machine OR Transfer Learning OR Learning, Transfer ) | 9813 |
| S1 | (MH "Machine Learning") | 7998 |
