# Supplementary material for: Machine Learning in the Prediction of Venous Thromboembolism: Systematic Review and Meta-Analysis
Source: J Med Internet Res. 2025 Dec 23;27:e77339. doi: 10.2196/77339 (PMC12724482; doi:10.2196/77339)
Supplement: Multimedia Appendix 2 [file jmir-v27-e77339-s002.doc]

**Multimedia Appendix 3. Risk of bias assessment**

| Author | Participants | Predictors | Outcome | Analysis | Summary |
| --- | --- | --- | --- | --- | --- |
| Liu S 2019 | Low | Low | Low | High | High |
| Nafee T 2020 | Low | Low | Low | Low | Low |
| Wang X 2020 | Unclear | Low | Low | Unclear | Unclear |
| Hou L 2021 | Unclear | Low | Low | Low | Unclear |
| Ryan L2021 | High | Low | High | High | High |
| Liu H 2021 | High | Low | Low | Low | High |
| Ryan L 2022 | Low | Low | High | Unclear | High |
| Lei H 2022 | Low | Low | High | High | High |
| Jin S 2022 | Unclear | Low | Low | Low | Unclear |
| Yan YD 2023 | Unclear | Low | Unclear | Low | Unclear |
| Wang X 2023 | Unclear | Unclear | Low | High | High |
| Wang KY 2023 | High | Low | High | Low | High |
| Shohat N 2023 | Low | Low | High | High | High |
| Sheng W 2023 | High | Low | High | Low | High |
| Qin L 2023 | Unclear | Low | Low | High | High |
| Papillon SC 2023 | Unclear | Low | Low | Low | Unclear |
| Ding R 2023 | Unclear | Low | Low | High | High |
| Hou T 2023 | Unclear | Low | Low | Low | Unclear |
| Nassour N 2023 | High | Low | Low | Low | High |
| Katiyar P 2023 | Low | Low | High | Low | High |
| Lin B 2024 | High | Low | High | Low | High |
| Liu L 2024 | Unclear | Low | Low | Low | Unclear |
| Wei C 2024 | Unclear | Low | Low | Low | Unclear |
| Wu X 2024 | High | Low | Low | High | High |
| Zhou H 2024 | High | Low | High | High | High |
| Chen X 2024 | High | Low | High | Low | High |
| Huang T 2024 | High | Low | High | Unclear | High |
