# Supplementary material for: Machine Learning in the Prediction of Venous Thromboembolism: Systematic Review and Meta-Analysis
Source: J Med Internet Res. 2025 Dec 23;27:e77339. doi: 10.2196/77339 (PMC12724482; doi:10.2196/77339)
Supplement: Multimedia Appendix 3 [file jmir-v27-e77339-s003.doc]

**Meta-Analysis**

**
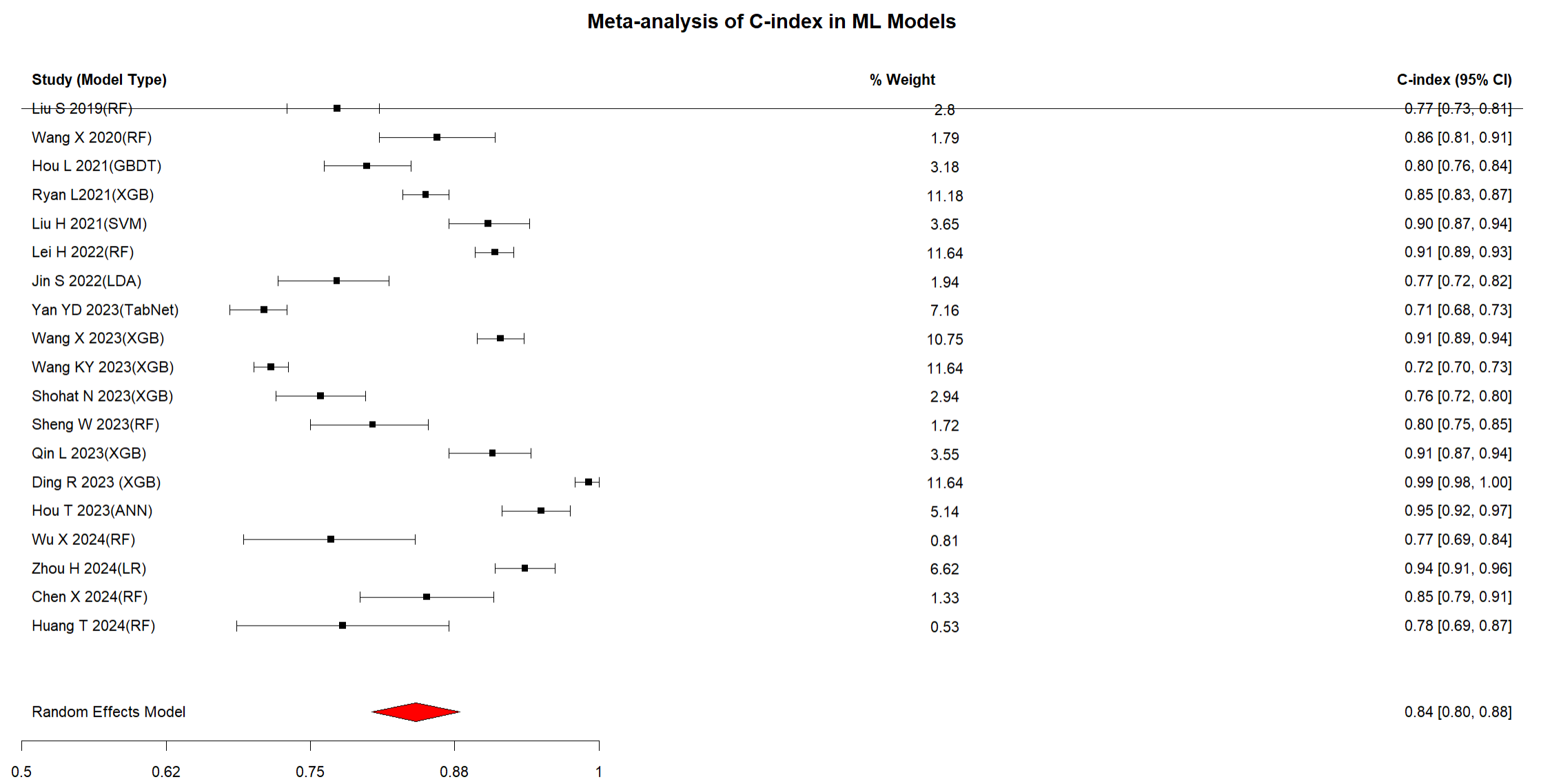
**

**Figure S1.** Concordance index forest plots of machine learning models for venous thromboembolism.

**Publication Bias**

In this study, The funnel plot analysis demonstrated a symmetrical distribution of Winsorized diagnostic odds ratios (DOR) across the precision spectrum (1/√(logESS) ranging from 0.5 to 0.9, with studies distributed proportionally across effective sample size (ESS) (*ESS*=(TP+FP+TN+FN)4×(TP+FP)×(TN+FN)​)categories (<200, 200-400, and >400) and showing no significant asymmetry (p = 0.067). Notably, the majority of studies fell within the smaller ESS categories (<200 and 200-400), while fewer studies were represented in the largest sample size category (>400).


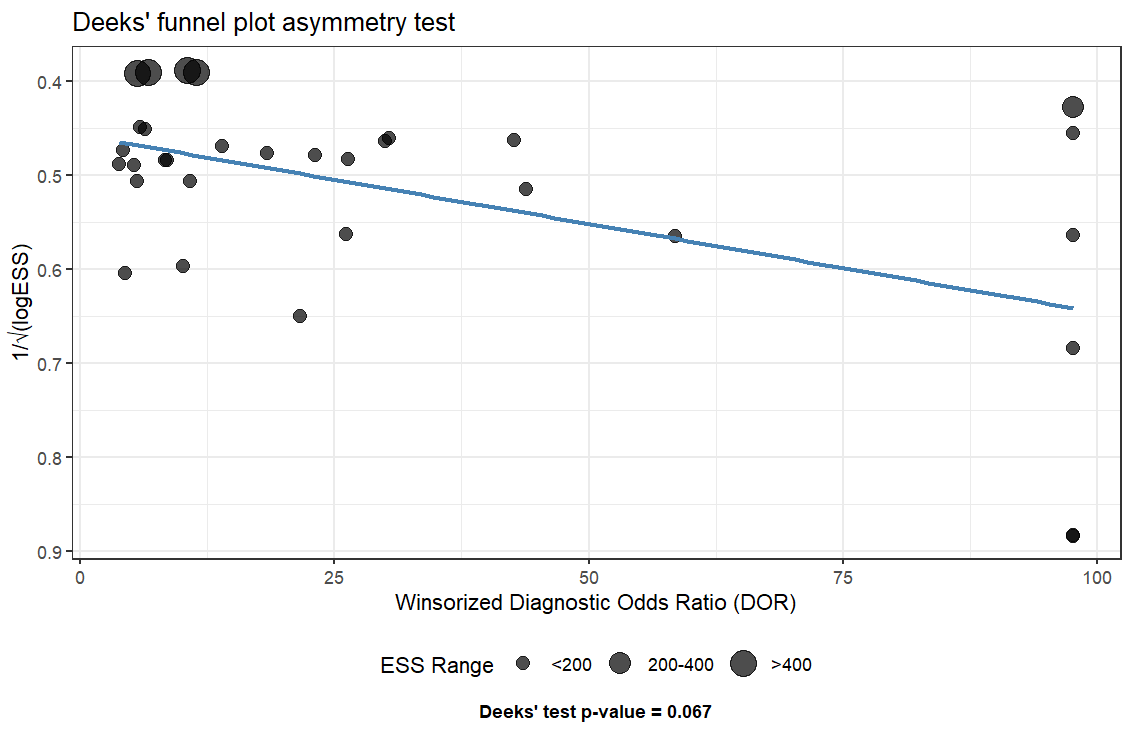


**Figure S2.** Funnel Plot Assessing Publication Bias in Meta-Analysis of Venous Thromboembolism (VTE) Risk Prediction Models.

**Subgroup Analysis**


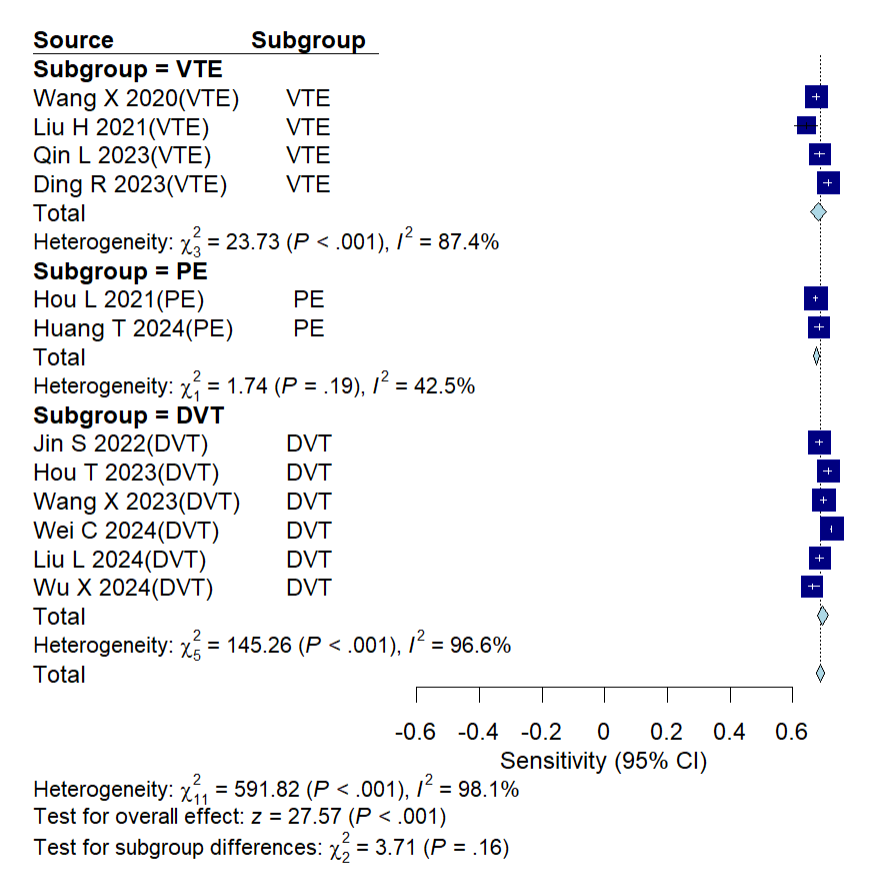


**Figure S3.** Venous thromboembolism subgroup forest plots of the pooled sensitivity for the diagnostic performance of machine learning.


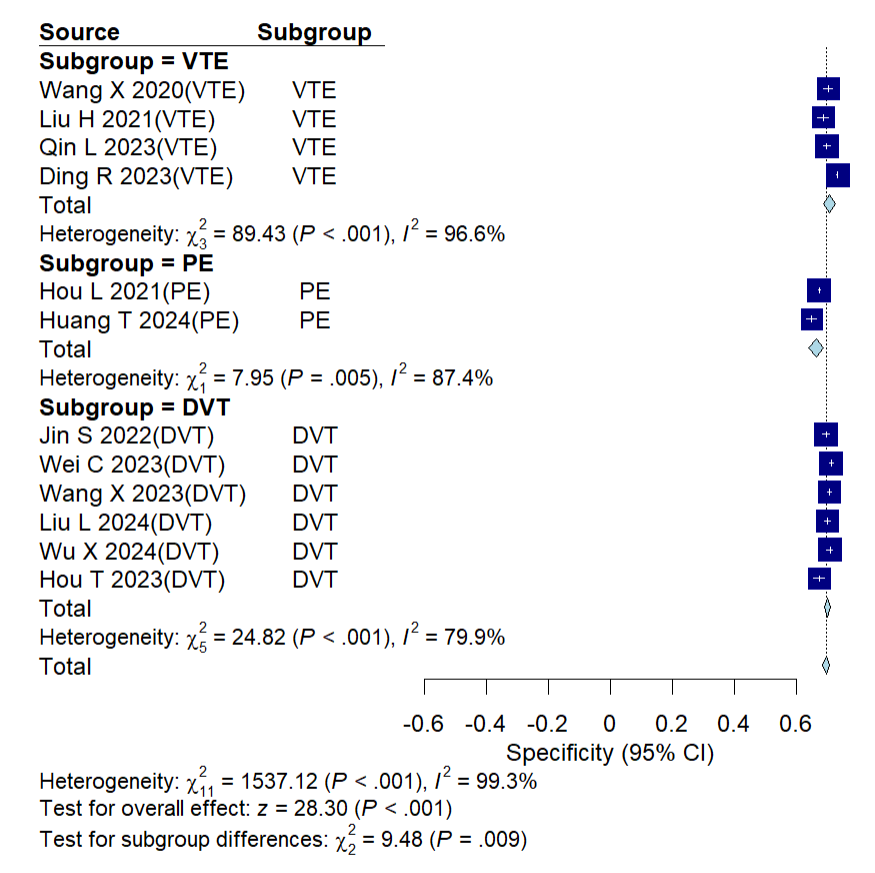


**Figure S4.** Venous thromboembolism subgroup forest plots of the pooled specificity for the diagnostic performance of machine learning.


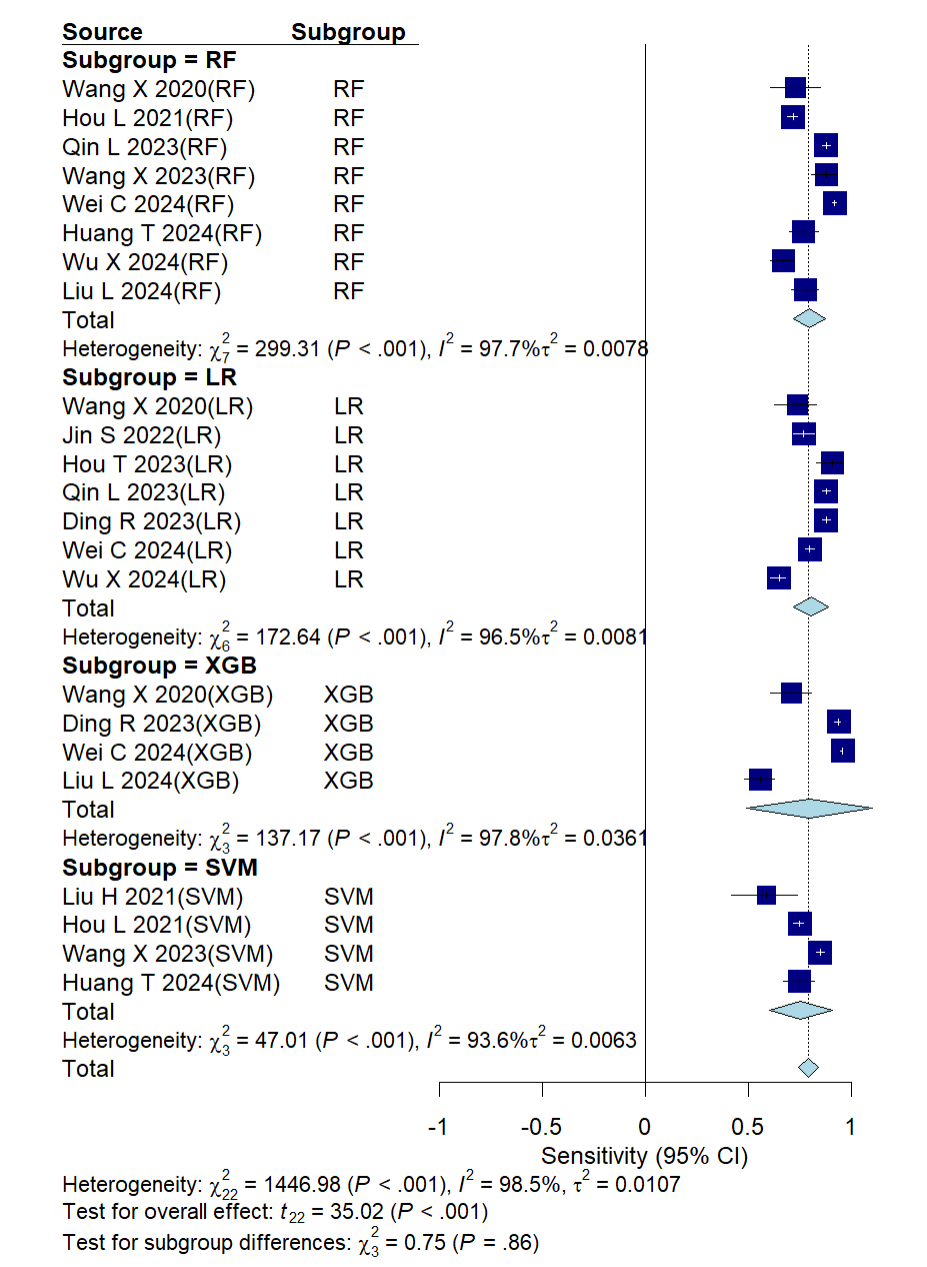


**Figure S5.** Machine learning model subgroup forest plots of the pooled sensitivity for the diagnostic performance for venous thromboembolism. The values and horizontal lines indicate pooled estimates with 95% confidence intervals (95% CIs).


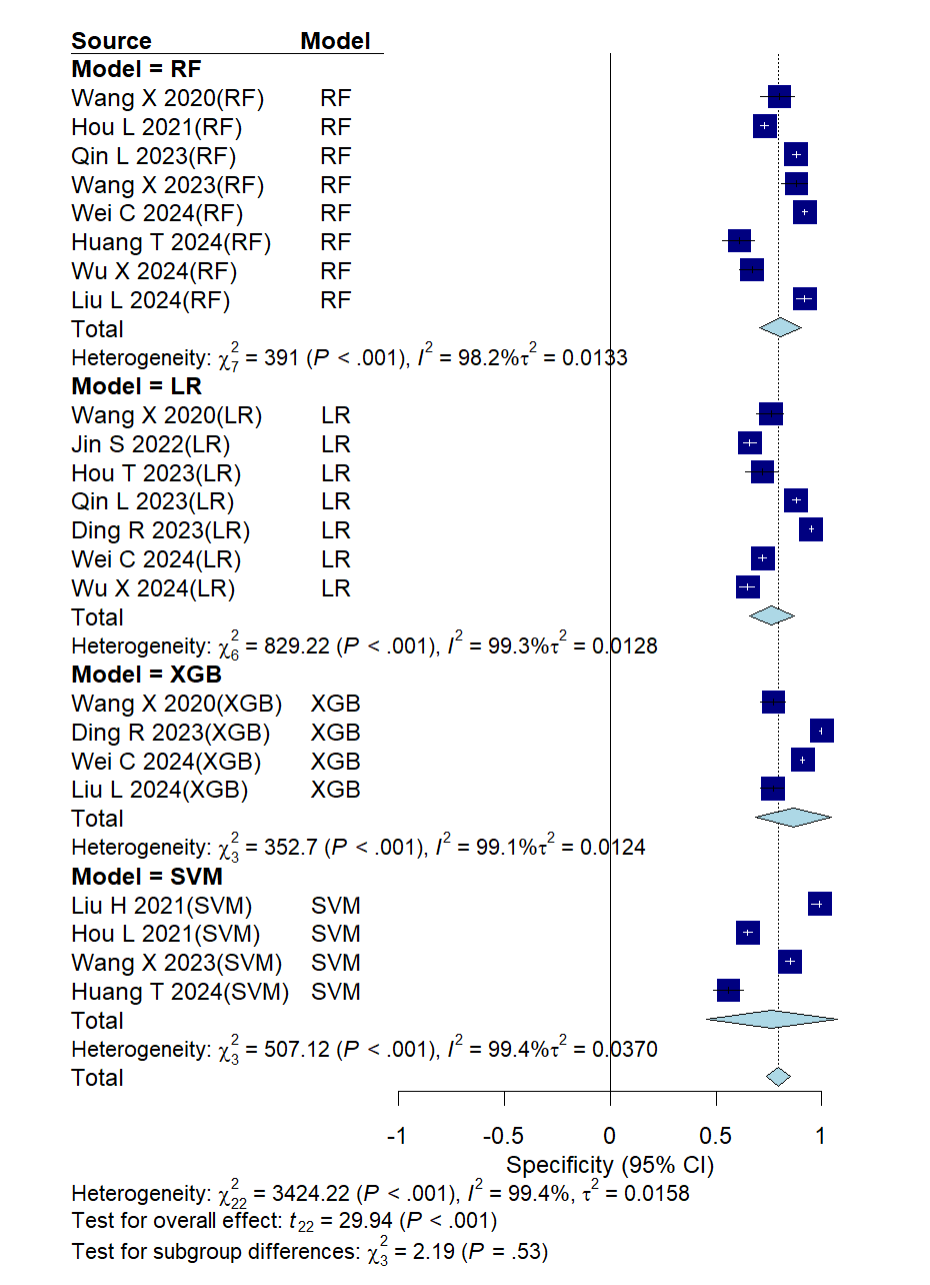


**Figure S6.** Machine learning model subgroup forest plots of the pooled specificity for the diagnostic performance for venous thromboembolism. The values and horizontal lines indicate pooled estimates with 95% confidence intervals (95% CIs).
