# Supplementary material for: Machine Learning in the Prediction of Venous Thromboembolism: Systematic Review and Meta-Analysis
Source: J Med Internet Res. 2025 Dec 23;27:e77339. doi: 10.2196/77339 (PMC12724482; doi:10.2196/77339)

**Multimedia Appendix 5. Frequency of predictors inclusion in machine learning models for venous thromboembolism based on the included studies.**


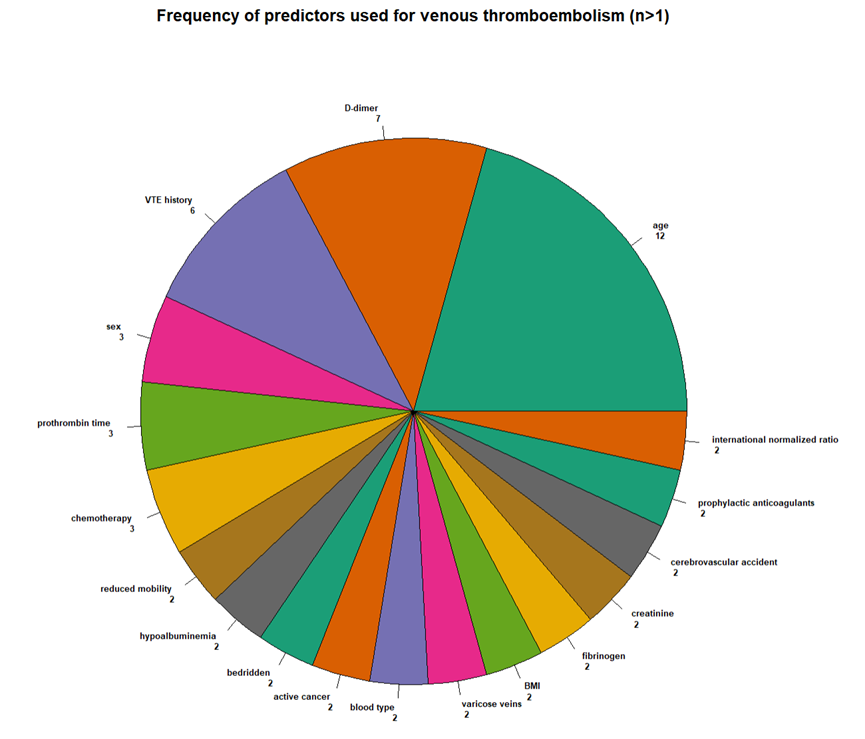

Supplement: Multimedia Appendix 4 [file jmir-v27-e77339-s004.doc]
