# Supplementary material for: Machine Learning in the Prediction of Venous Thromboembolism: Systematic Review and Meta-Analysis
Source: J Med Internet Res. 2025 Dec 23;27:e77339. doi: 10.2196/77339 (PMC12724482; doi:10.2196/77339)
Supplement: Multimedia Appendix 5 [file jmir-v27-e77339-s005.doc]

**Table 3.** Utilization of SHAP for Model Interpretation Across Included Studies

| Author | Utilization of SHAP |
| --- | --- |
| Liu S 2019[10] | none |
| Nafee T 2020[11] | none |
| Wang X 2020[12] | none |
| Hou L 2021[13] | none |
| Ryan L2021[14] | yes |
| Liu H 2021[15] | yes |
| Ryan L 2022[16] | yes |
| Lei H 2022[17] | none |
| Jin S 2022[8] | none |
| Yan YD 2023[18] | none |
| Wang X 2023[19] | none |
| Wang KY 2023[20] | none |
| Shohat N 2023[21] | none |
| Sheng W 2023[22] | yes |
| Qin L 2023[23] | yes |
| Papillon SC 2023[24] | none |
| Ding R 2023[25] | yes |
| Hou T 2023[26] | none |
| Katiyar P 2023[27] | none |
| Nassour N 2024[28] | none |
| Lin B 2024[29] | none |
| Liu L 2024[30] | yes |
| Wei C 2024[31] | none |
| Wu X 2024[32] | none |
| Zhou H 2024[33] | none |
| Chen X 2024[34] | none |
| Huang T 2024[35] | none |
